# The transcriptome of *Toxoplasma gondii*

**DOI:** 10.1186/1741-7007-3-26

**Published:** 2005-12-02

**Authors:** Jay R Radke, Michael S Behnke, Aaron J Mackey, Josh B Radke, David S Roos, Michael W White

**Affiliations:** 1 Department of Veterinary Molecular Biology, Montana State University Bozeman, MT 59717, USA; 2 Department of Biology and Penn Genomics Institute, University of Pennsylvania, Philadelphia, PA 19104, USA

## Abstract

**Background:**

*Toxoplasma gondii *gives rise to toxoplasmosis, among the most prevalent parasitic diseases of animals and man. Transformation of the tachzyoite stage into the latent bradyzoite-cyst form underlies chronic disease and leads to a lifetime risk of recrudescence in individuals whose immune system becomes compromised. Given the importance of tissue cyst formation, there has been intensive focus on the development of methods to study bradyzoite differentiation, although the molecular basis for the developmental switch is still largely unknown.

**Results:**

We have used serial analysis of gene expression (SAGE) to define the *Toxoplasma gondii *transcriptome of the intermediate-host life cycle that leads to the formation of the bradyzoite/tissue cyst. A broad view of gene expression is provided by >4-fold coverage from nine distinct libraries (~300,000 SAGE tags) representing key developmental transitions in primary parasite populations and in laboratory strains representing the three canonical genotypes. SAGE tags, and their corresponding mRNAs, were analyzed with respect to abundance, uniqueness, and antisense/sense polarity and chromosome distribution and developmental specificity.

**Conclusion:**

This study demonstrates that phenotypic transitions during parasite development were marked by unique stage-specific mRNAs that accounted for 18% of the total SAGE tags and varied from 1–5% of the tags in each developmental stage. We have also found that *Toxoplasma *mRNA pools have a unique parasite-specific composition with 1 in 5 transcripts encoding Apicomplexa-specific genes functioning in parasite invasion and transmission. Developmentally co-regulated genes were dispersed across all *Toxoplasma *chromosomes, as were tags representing each abundance class, and a variety of biochemical pathways indicating that trans-acting mechanisms likely control gene expression in this parasite. We observed distinct similarities in the specificity and expression levels of mRNAs in primary populations (Day-6 post-sporozoite infection) that occur prior to the onset of bradyzoite development that were uniquely shared with the virulent Type I-RH laboratory strain suggesting that development of RH may be arrested. By contrast, strains from Type II-Me49B7 and Type III-VEGmsj contain SAGE tags corresponding to bradyzoite genes, which suggests that priming of developmental expression likely plays a role in the greater capacity of these strains to complete bradyzoite development.

## Background

*Toxoplasma gondii *belongs to the phylum Apicomplexa, which comprises a diverse group of protozoa, considered to share much of the biology underlying obligate occupation of a host cell and responsible for disease in a range of host species. *Toxoplasma *is distinct from most members of the large coccidian family contained in this phylum owing to the exceptional number of animals that are able to serve as host including virtually all warm-blooded animals. While *T. gondii *completes the definitive life cycle in a single animal host (feline), the capacity of oocysts (shed from the feline host) as well as tissue cysts to infect multiple hosts has enabled *T. gondii *to increase the host range for the intermediate life cycle. This rare modification to the heteroxenous (two host) life cycle is thought to have occurred relatively recently and may be responsible for the expansion of this parasite to nearly every continent [1]. Parasite transmission via the oocyst stage has resulted in epidemics of human toxoplasmosis [2-6] and widespread infections of livestock that can also lead to human infections through the consumption of tissue cyst-contaminated food [7,8]. Together, oocyst and tissue cyst sources contribute to rates of human exposure such that the risk of infection in the U.S. is one in three by age 50 (25% for >20 yrs of age [9]; and nearly 100% by the end of childhood in other parts of the world [10]).

Given the importance of *Toxoplasma *infections to human populations, understanding developmental mechanisms leading to tissue cyst formation is critical for ultimately controlling transmission and chronic disease. Based on cat bioassays, tissue cysts are first detected in mouse tissues approximately one week from the time of oral inoculation of oocyst (containing sporozoites) or tissue cyst material (containing bradyzoites) [11,12]. The invariant course of *T. gondii *primary infections in animals suggests that developmental mechanisms initiated by either the sporozoite or bradyzoite stage are similar and are likely the consequence of an unfolding parasite genetic program. Studies of sporozoite- and bradyzoite-initiated development *in vitro *[13,14] support this view, as parasites emerging from infections of human foreskin fibroblasts (HFF) follow a defined course of development evident by nearly synchronous changes in growth and stage-specific gene expression that result in the emergence of bradyzoites 7–10 days later [13,14]. The key to this developmental pathway in *T. gondii *may lie in a shift to slower growth that occurs following a limited number of divisions in sporozoite-infected cultures, and is well documented in all studies of bradyzoite differentiation [13-17]. The link between cell cycle mechanisms and bradyzoite development is unknown, but is characterized by a transient slowing of S phase that leads to mature bradyzoites, which possess a uniform genome content consistent with cell cycle arrest in G1/G0 (1N DNA content) [13,14]. These studies suggest that a developmental timer system in primary *T. gondii *infections (oocyst or tissue cyst) may regulate tissue cyst development in the intermediate host.

The frequency of bradyzoite switching (tachyzoite to bradyzoite, bradyzoite to tachyzoite) varies among *Toxoplasma *isolates and may influence the level of parasite expansion in animals. As such, defining the changes in gene expression that accompany this development pathway is important for understanding the molecular events that contribute to chronic as well as acute disease. EST projects have been undertaken in *Toxoplasma *[18,19] for the purpose of gene discovery, but have also confirmed earlier studies that first demonstrated that novel gene expression is associated with the major intermediate life cycle stages [20,21]. EST sequencing has led to the development of a limited *Toxoplasma *cDNA array [22-24] that focused on tachyzoite-bradyzoite transitions in cell culture models of bradyzoite differentiation [22] and explored gene expression in mutants that are unable to differentiate [23,24]. It is not possible given the small size of these arrays (~600 genes) to draw global themes about *Toxoplasma *gene expression; however, these studies are important in that they confirm that changes in mRNA levels correlate with the expression of known bradyzoite protein antigens (supporting transcriptional mechanisms) and provide some evidence that a hierarchal progression of gene expression may govern development in this parasite [23]. At present, the *Toxoplasma *genome has been sequenced to 10X-coverage of the Me49B7 Type-II strain [25] with plans to extend coverage to Type I and III strains. Even in the absence of whole genome sequence for all three lineages, a complete map of the parasite transcriptome will allow us to begin analysis of development and inter-strain variation.

In this paper, we report a comprehensive investigation into the whole cell changes in the levels of mRNAs occurring during progression of parasite populations through the *T. gondii *intermediate life cycle. Additionally, we have examined laboratory strains representing the three major genotypes and demonstrate that specific patterns of gene expression are uniquely shared between laboratory strains and the primary parasite stages characteristic of specific transitions in the *T. gondii *intermediate life cycle.

## Results and discussion

### Whole cell analysis of gene expression in the protozoan, *Toxoplasma gondii*

We have previously reported that the sporozoite or bradyzoite stages traverse several growth transitions that are accompanied by specific alterations in gene expression leading to bradyzoite development [13,14,26]. To define how the parasite transcriptome changes during this developmental progression, we have constructed and sequenced SAGE libraries [27] from: (i) oocyst populations (containing mature sporozoites prior to release from the oocyst), (ii) parasites emerging from the sporozoite-infected cell at Day-4 post-inoculation, (iii) parasites rapidly growing in the second host cell (Day-6 post-sporozoite infection), (iv) parasites immediately following the growth shift at Day-7 post-inoculation, and (v) from slowly growing mixed tachyzoite-bradyzoite populations at Day-15 post-sporozoite inoculation. A library was constructed from primary VEG parasites (passaged >5 times from the initial sporozoite inoculation) that were shifted more fully into bradyzoite development by alkaline stress (RNA was harvested 72 h post-alkaline shift and the library was designated as "pH-shift"). Additional libraries were constructed from three laboratory-adapted strains representing the major genotypic variants of *Toxoplasma *(Type I-RH, Type II-Me49B7 and Type III-VEGmsj). Thus, six libraries were constructed from progressive 'snap-shots' of the sporozoite to bradyzoite developmental pathway, while the three laboratory strains represent tachyzoite forms that display a range of virulence and developmental capacity [13]. All nine SAGE libraries were constructed by template switching [28] using oligo-d(T) priming and each library was sequenced to a depth of greater than 20,000 tags. In all, and after sequence error correction, 290,062 tags were obtained of which 38,263 were unique with 90% of the tags matching sequence obtained from *T. gondii *genome reference strain Me49B7 (Table 1). The nearly 10% of SAGE tags that were not a perfect match to the genome reference either had lower average frequencies or represented tags containing single nucleotide polymorphisms. The RNA content of asynchronously growing *T. gondii *parasites (RH strain) has been experimentally determined to average ~0.3 pg of total RNA per cell. Thus, in comparison to the unicellular yeast [29], which is similar in size and RNA content (0.44 pg/cell [30]), the *Toxoplasma *SAGE project is predicted to exceed 4 × coverage of the genes expressed in the intermediate life cycle (>97% of mRNAs represented by at least one tag [29]). Access to this dataset (ALL in Table 1) is available at TgSAGEDB [31]. A low redundancy dataset designated 2 × 2 was also produced for various analyses that eliminated SAGE tags with >2 matches to genome sequence (based on the 14 bp tag that includes the CATG and 10 bp cDNA sequence) and tags found only once in the combined libraries. This dataset comprises 202,472 tags or 69.8% of the corrected tags that matched the *Toxoplasma *genome sequence (Table 1). All analyses were conducted with the 2 × 2 set unless otherwise stated.

**Figure 1:**
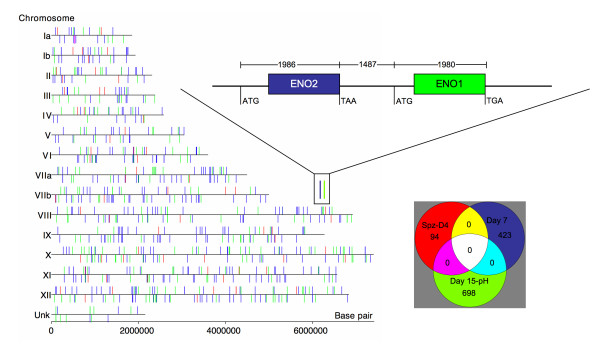
**Chromosome landscape of mRNA expression in *Toxoplasma***. SAGE tag distribution with respect to three classes of co-regulated mRNAs are indicated in each chromosome assembly [32]; sporozoite- (red), Day-7 (blue) and Day-15/pH-shift (green) specific. Transcriptional orientation for each tag plotted is indicated by the up (forward) or down (reverse) direction of the corresponding mark. Inset Venn diagram shows the overall number of unique tags represented and demonstrates that there is no overlap in the expression of these classes of mRNA. The expanded diagram demonstrates the differentiation expression of the tandem head-to-tail sequences encoding enolase 1 and 2.

The average density of SAGE tags in the fourteen *Toxoplasma *chromosome assemblies [32] was remarkably consistent. We observed a SAGE tag on average every 6,003 bp with 5,407–6,788 bp between SAGE tags across all assemblies. Taking into account the average nucleotide distance between SAGE tags, and the predicted gene length of ~4,486 bp (Mackey and Roos, unpublished results), we estimate that intergenic regions are ~2,000 bp, congruent with the 3,404 bp proposed from the genome project. This finding validates the global coverage of the expressed genome by the SAGE project and is consistent with a >4-fold coverage of the parasite mRNA pools. Regions in the chromosome assemblies lacking EST or SAGE tags vary in length with those exceeding 10,000 bp between tags totaling nearly 16% (~10 MB) of the *Toxoplasma *genome sequence. Annotation of the non-expressed genomic sequences (as defined here) reveals fewer general BLAST hits (3.8%) when compared to regions containing EST or SAGE tags (~20%), and the non-expressed regions are not enriched for known apicomplexa gamete or merozoite (primarily from *Eimeria *species) genes, although gene expression in these developmental stages are not well characterized in *T. gondii*. Thus, more than half of the *Toxoplasma *genome (63 Mb) is occupied by gene-transcription units that reflect gene expression in the oocyst and intermediate life cycle stages, and the relatively close spacing of genes that results from this organization suggests that, as in yeast, transcriptional mechanisms in *Toxoplasma *are probably more gene-proximal than in higher eukaryotes [33]. Overall, developmentally co-regulated mRNAs and mRNAs encoding proteins from specific biochemical pathways and mRNAs representing different abundance classes are distributed across all *Toxoplasma *chromosomes, indicating that gene transcription is not organized into polycistronic units but, as observed in *Saccharomyces *[29], is dispersed throughout the genome (chromosome distribution of abundant, apicomplexa-specific and stage specific tags are shown in Fig. 1). An examination of several clusters of paralogous genes that do occur in the *Toxoplasma *genome demonstrated that mRNA expression in these clusters was divergent. For example, enolase 1 and 2 [19], SRS9/BRS4 [22] and SAG4A/SAG4.2 [34] occur in tandem head-to-tail configurations that are closely spaced (<1,500 bp for the enolases; Fig. 1 inset). Despite this proximity, individual genes in each pair are distinctly regulated. ENO2 and BRS4 mRNAs are expressed in tachyzoites while enolase 1 and SRS9 and SAG4.2(p18) (no tags were observed for SAG4A) encode bradyzoite-specific mRNAs (reviewed in [35]). Taken together, these observations suggest that transcriptional activation or repression in *Toxoplasma *is effective over a limited sequence distance and is consistent with a model of co-regulation that involves gene-specific trans-acting factors.

### *Toxoplasma *mRNA pools have a distinctive composition with respect to complexity, stage-specificity and anti-sense transcripts

To estimate gene number represented by the SAGE project, we assembled genome sequence flanking SAGE tags from the 2 × 2 set (8,188 unique tags) using 2,000 bp 5'-upstream of each tag (cap3 assembly). This bracket was conservative with respect to gene overlap given the >4,000 bp average gene size. Combined, contigs and singletons for this assembly comprise 6,372 sequences, which is proportional in size to the total set of 7,793 genes predicted for the whole genome (Mackey and Roos, unpublished results) given that parasite stages from the feline host were not included in the SAGE project. Analysis of the 2 × 2 dataset demonstrated that ~25% of the unique tags annotated with sequence from the NCBI-nr database (representing 50% of the 2 × 2 tag counts) with an average frequency of 50 tags per annotated sequence (Table 1). When comparing this set to the unassembled ESTs (125,769 ESTs) [36], 44% of the unique SAGE tag sequences matched an EST (80.6% of total tag counts). Roughly 50% of unique SAGE tag sequences (20% by frequency) do not match any EST, likely reflecting the 5' versus 3' preference of these methods as well as the relative greater depth of sequencing in the SAGE project.

**Figure 2:**
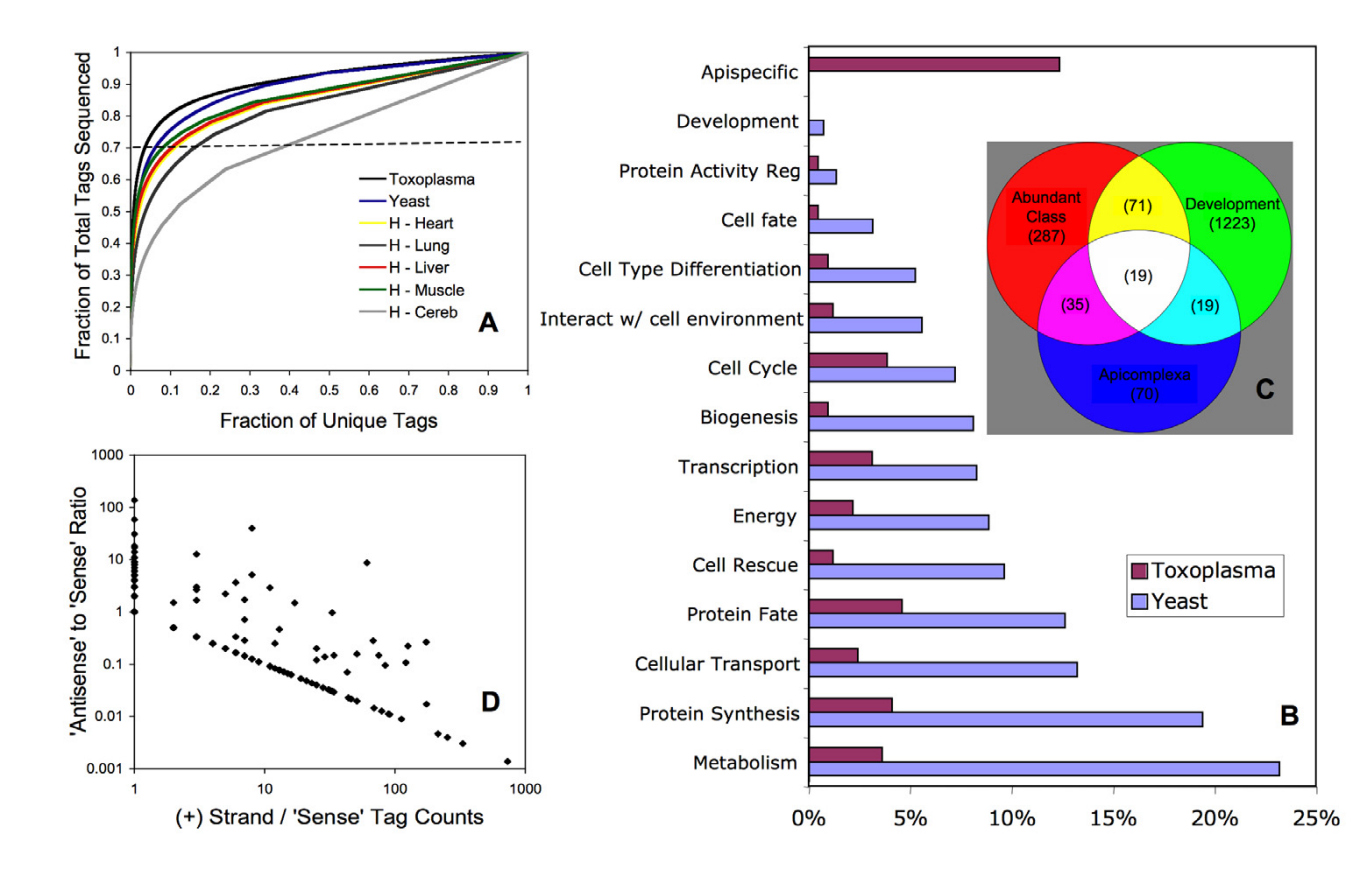
***Toxoplasma *mRNA pools have a distinctive composition**. [A] The mRNA abundance classes in *Toxoplasma *were characterized in comparison to SAGE datasets obtained from yeast, and from human libraries constructed from heart, lung, liver, muscle and cerebellum. For each comparison, unique SAGE tags are plotted in reverse order as a fraction of total unique tags in the library beginning with the highest frequency tags (X-axis). These results were plotted against the concentration for each unique tag, defined as the fraction of the total SAGE tag concentration for that library (Y-axis). [B] Abundant SAGE tags corresponding to highly expressed mRNAs fall into a number of functional classes [97] with unknown genes (not displayed here) and apicomplexa-specific genes involved in parasite invasion the dominant categories for *Toxoplasma*. [C] Venn diagram displaying SAGE tags belonging to three distinct classes; high abundant tags in the 2 × 2 dataset (red), tags uniquely up- or down-regulated in one of the six developmental libraries (green), and Apicomplexa-specific SAGE tags encoding proteins found in dense granules, rhoptries, micronemes and on the parasite surface (blue). [D] Anti-sense transcription in relation to SAGE tag abundance. The ratio of antisense versus sense was determined at each genomic locus using GeneFinder gene prediction dataset derived from Me49B7 genome sequence. Levels of 'antisense' as fractions of the 'sense' transcription were quantified for each gene prediction dataset and plotted against the frequency of the sense tag. Similar plots were obtained using three other gene predictions from TwinScan, TigrScan and Glimmer (not shown).

The mRNA abundance classes revealed by the 2 × 2 dataset were partitioned into 5% high abundant (>100 frequency per tag), 25% moderate abundant (11–90 frequency), or 70% low abundant (2–10 frequency). An estimated 412 tags (representing 412 unique genes) constituted the high abundant class in the 2 × 2 set (gene list is available at TgSAGEDB [31]). In comparison to yeast and human cells, *Toxoplasma *appears to devote a greater proportion of its transcriptional energy to a relatively lower number of genes. As few as 3.1% of the total unique tags from the full corrected SAGE dataset (290,062 tags) encompassed 70% of all the tags (and transcripts) generated in the SAGE project (Fig. 2A). By contrast, 6.6% and 8–36% of the unique SAGE tags produced from yeast [37] and human [38] mRNA sources, respectively, composed 70% of the normalized SAGE datasets (Fig. 2A) – demonstrating that by comparison *Toxoplasma *mRNA pools are notably less complex. The compositions of highly expressed mRNA pools in *Toxoplasma *were different from yeast (shown in Fig. 2B) with apicomplexa-specific genes (13% of the unique 412 tag set; Fig. 2C) and unknown mRNAs the single largest categories (64.5% for *Toxoplasma *versus 27% for yeast, not graphed). Based on total frequency, >1 in 5 parasite tags corresponds to transcripts encoding dense granule, microneme and rhoptry proteins or surface antigens. In general, apicomplexa-specific genes have simple genomic structures containing few if any introns. The structure of these genes may be a consequence of their high expression, as intron number and length is known to influence mRNA expression levels negatively in other eukaryotes [39-41]. While there are relatively fewer non-apicomplexa genes in the *Toxoplasma *abundance class, the genes represented appear to reflect shared eukaryotic biology (Fig. 2C), supporting the view that individual mRNA levels are influenced by evolutionary selection for cellular fitness [39]. Genes encoding proteins that function in metabolism and protein synthesis and fate were highly expressed in all libraries examined including human and, not surprisingly, also in microarray datasets from *Plasmodium *(not shown here [42]). For example, the glycolytic enzymes glyceraldehyde 3-phosphodehyrogenase (GAPDH), enolase and fructose 1,6 bisphosphate were found in the abundant class of all three eukaryotes. Thus, selection for the high expression of genes functioning in parasite invasion and transmission appears to have had equal footing with the conserved pathways of protein synthesis and metabolism in the evolution of gene expression in this protozoon. It is worth noting that the functional analysis of the top 25 assemblies from the EST dataset shows clear overlap with the above SAGE analysis [43].

The unique composition of *Toxoplasma *mRNA pools that are populated with apicomplexa-specific genes may be mirrored by the parasite transcriptional machinery, and while the full repertoire of general and specific transcription factors has not been determined for *Toxoplasma*, it has been noted that sequence divergence of transcriptional activator proteins is strongly correlated to evolutionary distance [44]. This view is supported by the very recent discovery that general transcription factors in *Plasmodium *are highly divergent when compared to their counterparts in the crown group of eukaryotes [45,46].

Approximately 39% of SAGE tags from VEG developmental stages show a > 2.5-fold change in frequency in at least one library indicating that mRNA regulation is very active in this parasite. Changing mRNA levels were observed across all abundance classes, indicating that far fewer genes are constitutively expressed in these parasites. For example, only 37% of the 412 high abundant class mRNAs are constitutively expressed across all the developmental libraries and nearly a quarter (22%) of high abundant tags are stage-specific (Fig. 2C). Global comparisons of SAGE tags from VEG primary libraries (sporozoite, Day-4, -6, -7, -15, pH-shift libraries) demonstrate the unique changes in the mRNAs across development as standard correlations vary between 0.0229 to 0.32000 among these datasets (Table 2), with the exception of the comparison between sporozoite and Day-4 libraries (sporozoite vs. Day-4 r = 0.728). SAGE tags uniquely associated with each *Toxoplasma *developmental library encompass ~18% of the total 2 × 2 SAGE tags and range from 1.5–5.3% of the tags in each library. Lists of stage-specific genes identified in these studies may be accessed at TgSAGEDB [31]. It is significant that our results parallel microarray studies in *Plasmodium *where developmental mRNA expression is also largely stage-specific [47].

The unique tag sequence and polarity with respect to mRNA sequence allows for the determination of global sense and antisense transcripts by placing tags within the orientation of predicted open-reading-frames within the genome. Similar analyses using SAGE tags derived from *P. falciparum *mRNAs identified antisense transcripts (12%) that were inversely related to the nearest sense transcription [48]. Here, antisense transcription has been independently confirmed [48], although the function of these transcripts has yet to be established. In order to determine whether antisense transcription was conserved in *Toxoplasma*, we performed a global analysis of tag frequency (using tags that matched once in the genome) and orientation in comparison to four different predicted gene sets obtained from annotation of the *Toxoplasma *genome [25]. The range of antisense transcription obtained with each gene set was similar, with an average of 21.5% of the total SAGE tags observed to encode a predicted opposite strand transcript. As was found in *P. falciparum *[48], there was a strong inverse relationship between the frequency of antisense transcripts and the level of sense transcription detected within the predicted gene sequence, and as such, the higher a sense transcript was expressed, the lower we observed the level of potential antisense transcription (Fig. 2D). Thus, this unusual feature of transcription in *P. falciparum *extends to a distantly related member of the Apicomplexa phylum and opens the door to studying this important biological mechanism, taking advantage of the *Toxoplasma *experimental model.

### SAGE tags contain single nucleotide polymorphisms (SNPs) and reflect differential polyA+-choices

**Figure 3:**
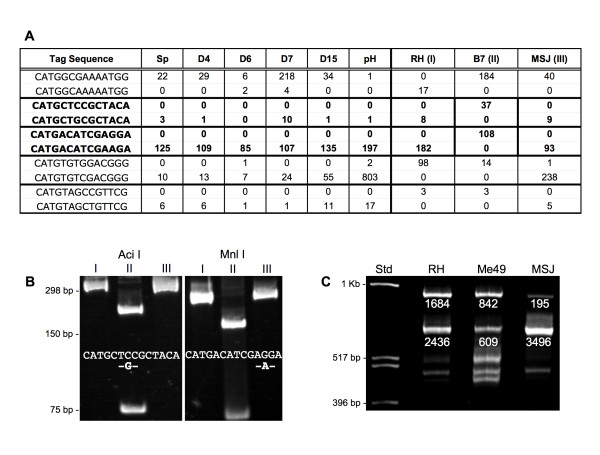
**Single nucleotide polymorphisms and differential polyA+-choices are reflected in SAGE tag datasets**. [A] Screening for reciprocal frequency patterns of nearest neighbor SAGE tags identifies potential strain-specific polymorphisms within the SAGE library datasets. Out of more than 400 tags containing SNPs, a selected set of five SNP-containing SAGE tags that represent all known biallelic patterns are shown [31] (I, III versus II; I, II versus III; II, III versus I). SAGE tag frequencies from all nine libraries are listed; VEG post-sporozoite SAGE libraries; Sp = sporozoite, D4 = Day-4 post-sporozoite infection, D6 = Day-6, D7 = Day-7, D15 = Day-15, pH = alkaline shifted parasites. [B] RFLP analysis was performed to verify strain-specific polymorphisms in two selected SAGE tags. Primers were designed from sequence flanking each SAGE-tag position within *Toxoplasma *genomic sequence. PCR fragments were amplified from genomic DNA from each of the three genotypic strains, purified, and digested with the appropriate restriction enzyme. Molecular mass standards are displayed. The sequence for Type II SAGE tag CATGCTCCGCTACA is found on chromosome V-1,674,592 and contains a mutation in the Aci I binding site. The Type II SAGE tag CATGACATCGAGGA is located on chromosome X-2,748,371 and within the coding region for the *Toxoplasma *homolog of SUL1 (AAF76883.1). A single nucleotide change in Type I and III SAGE tag occurs within the binding site for restriction endonuclease Mnl I. Note that in each case shown the sequence variation is biallelic; I, III versus II. [C] SAGE tag clusters indicate strain-specific differences in polyA+-addition. A 3'RACE reaction was performed on Type I (RH), Type II (ME49B7) and III strain (VEG) total RNA using a GRA7-specific primer in conjunction with a modified oligo-d(T) primer. The distance of the Nla-III site (anchor site for each SAGE tag) from the GRA7-specific primer was correlated to the 3'-RACE products and the 3'-RACE products were verified by cloning and sequencing. The corresponding tag frequencies for the top two 3'RACE products are indicated below the band in each strain. Four tags with similar frequency were obtained from the Type II library that correlate with Type II 3'-RACE products; 3'-RACE 951 bp product = tag with 842 frequency, 600 bp = 609 tags, 520 bp = 136 tags, 409 bp = 401 tags. The 951 bp and 600 bp 3'-RACE products were dominant in RH and VEG strains.

A significant number of SAGE tags with relatively high frequency did not match the *Toxoplasma *genome sequence from the Type II Me49B7 strain. Given the bias of the SAGE dataset to mRNA pools from the Type III VEG strain it was possible that some of these tags might reflect strain-specific polymorphisms. Using a nearest neighbor analysis, we have related SAGE tags containing single nucleotide differences such that tags containing SNPs might be identified on the basis of a reciprocal pattern of expression (access to the complete SNP data can be obtained at TgSAGEDB [31]). A total of 446 VEG strain tags with a ≥ 5 frequency did not have a match in the Me49B7 genome sequence or the Me49B7 SAGE library. These tags are likely real in view of the estimate of SNPs in the EST database (3 SNP per 1,000 bp of cDNA sequence; Jon Boyle, personal communication), which predicts ~400 VEG tags may contain nucleotide polymorphisms. Five representative SAGE tags containing putative SNPs and their respective library frequencies are displayed in Figure 3A and include examples of all three biallelic patterns (I vs. II,III; II vs. I,III; III vs. I,II) observed in natural isolates of *Toxoplasma *[32]. Where the nucleotide change occurred within a unique restriction site (in the 14 bp SAGE tag), we were able to verify specific SNPs by RFLP analysis. Figure 3B demonstrates that the nucleotide variation identified in Type II versus Type I and III SAGE tags by neighbor analysis was confirmed by the presence of restriction endonuclease sites only in the Type II genomic sequence.

The extent of differential splicing or alternative termination of mRNAs in *Toxoplasma *is largely unknown. Alternate splicing of myosin [49] and hypoxanthine-xanthine-guanine-phosphoribosyltransferase [50] transcripts are examples where protein isoforms arise as the result of these mechanisms. The structural analysis of dihydrofolate reductase-thymidylate synthase (DHFR-TS) mRNA expression represents the only case where sites of polyA+-addition have been mapped [51]. The SAGE datasets have the potential to reveal significant information about alternate transcripts provided Nla-III sites (the anchor site for all SAGE tags in these libraries) are available to discriminate mRNA species. To explore the question of PolyA+-choice, we evaluated the clustering of SAGE tags using a 500 bp sequence bracket to gather genome proximal tags around each unique tag. Nearly two thirds of SAGE tags (61%) had no second tag within the 1 kbp sequence window while 20.6% had a second tag and 18.3% had ≥ 2 tags. The largest cluster comprised 10 tags. Interestingly, the tag frequency distributions of specific clusters sometimes varied significantly indicating that the underlying mRNA transcripts might be stage or strain specific. To test whether SAGE tag clusters might reflect differential PolyA+-site addition, we compared the specificity and relative ratio of cDNA fragments generated from 3'-RACE reactions to the nearest corresponding SAGE tag frequency of dense granule protein, GRA7. GRA7 shows a distinct pattern of tags that differentiates the three canonical *Toxoplasma *strains (Fig. 3C). RACE products generated from these RNA sources match reasonably well the relative SAGE tag patterns with Type I mRNA, yielding two major 3'-RACE products, and Type II RNA and Type III RNA, producing multiple or single RACE products respectively. Sequence analysis of the GRA7 3'-RACE cDNA fragments from all three strains confirmed that they were derived from GRA7 mRNA and correspond to authentic poly-adenylation sites in the GRA7 3'-UTR. Thus, the multiple SAGE tags for GRA7 are not the result of partial digests in SAGE library construction, but reflect true sites of polyA+-addition that are differentially regulated.

### Parasites emerging from the sporozoite-infected cell retain significantsporozoite gene expression

**Figure 4:**
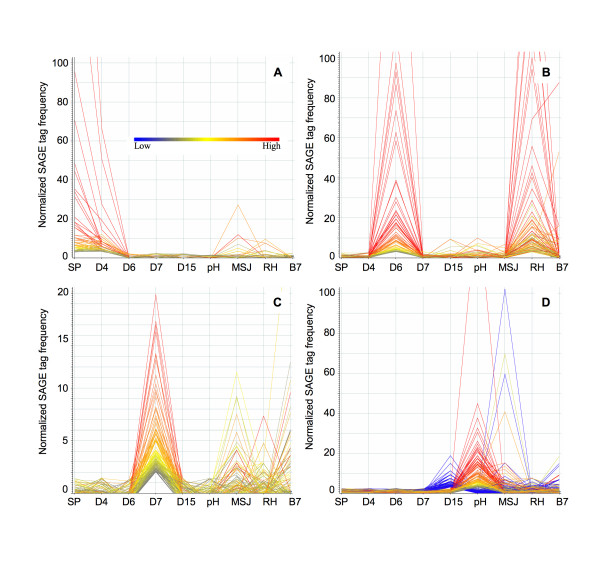
**Groups of co-regulated mRNAs are associated with the major developmental transitions**. Stage-specific gene expression as defined by normalized fold-change within the six developmental libraries was determined without regard to the level of expression in the laboratory-adapted strain libraries. SAGE tag clusters for the combined sporozoite and Day-4 post-sporozoite infection, Day-6, Day-7 and Day-15/pH-shifted and their respective plots were generated using GeneSpring 7.2 software. Similar distributions were obtained using standard k-means clustering (not shown). Note that expression of each SAGE tag within the four co-regulated sets was plotted for all nine libraries including the laboratory strains with expression color-coded by the level in the relevant developmental libraries. [A] Parasites emerging from sporozoite-infections at Day-4 post inoculation possessed high levels of sporozoite-specific gene expression (0.728 correlation between these libraries) that were nearly all down-regulated by Day-6 post-sporozoite infection and beyond. Note that with the exception of a few SAGE tags in the Type III (VEGmsj) library there is little expression of these genes in the laboratory strains. [B] Genes specifically expressed >3-fold in Day-6 post-sporozoite SAGE libraries (120 total) were also uniquely elevated in the Type I-RH library suggesting these parasites have a common gene expression that may reflect shared biology. Note that the expression of these genes is largely exclusive to RH and Day-6 and that the levels of expression are remarkably similar in each library. [C] Parasites post-growth shift at Day-7 (15 h doubling) represent a developmentally unique stage positioned at the initiation of bradyzoite development. Fully 5% of the total SAGE tags expressed in this stage showed regulation (15 % difference between Day-7 and Day-6) with relatively few known bradyzoite-specific genes expressed at this stage. Note that there was considerable shared expression of Day-7 SAGE tags with the laboratory strains, although expression levels showed higher or lower expression in lab strains as reflected in the color scale of individual curves that were fixed by the Day-7 levels in this gene plot. [D] Based on SAGE tag frequencies, 698 genes were found to be expressed >3-fold higher in the Day-15/pH-shift libraries. Genes identified in this set included the known bradyzoite genes BAG1, LDH2, SAG4.2, enolase 1, cyst-wall protein and a novel bradyzoite-specific NTPase. A number of the SAGE tags contained in this cluster were also elevated in the laboratory strains VEGmsj and Me49B7, while the RH strain showed little or no expression of the tags from this gene set as was found for the other developmental libraries including Day-6.

To determine whether distinct mRNA pools could be correlated with each sampled time point across development (e.g. sporozoite and Days-4, -6, -7 and -15 post-sporozoite inoculation and the pH-shift library), we compared each library with itself, and then separately with each of the other libraries in the series, and generated a standard correlation coefficient for normalized tag ratios (Table 2). Evaluation of *r *for each subsequent comparison demonstrates that the mRNA pools for each of these populations were essentially unrelated (*r *= 0.0229 to 0.32, Table 2). It is significant that Day-6 and Day-7 mRNAs were the most distinctive in the developmental pathway (see Table 2) when compared with both early and late development; and also that these two populations are even further differentiated from one another (r = 0.0604, see Table 2). This distinctiveness likely reflects the position in the pathway between rapidly-growing parasites at Day-4 and the full emergence of bradyzoites – two distinctly different phenotypes with respect to both growth and development. It was also evident that Day-7 and Day-15 mRNAs are more similar to one another than any two libraries outside of the sporozoite and Day-4 libraries discussed below (*r *= 0.32), however, mRNAs are distinctly different when the Day-7 is compared with the pH-shift library (r = 0.0888). This difference suggests that the mRNA pool represented in the pH-shift library is consistent with a further progression into the developmental pathway leading to mature bradyzoites. In early development, oocyst and Day-4 mRNA pools were found to be strikingly similar in content and expression level (*r *= 0.728). From the unique SAGE tags contained in sporozoite and Day-4 2 × 2 dataset, 2,259 tags were found to be expressed in both libraries within a ± 2-fold level of expression (95% of the tags expressed in the combined libraries). As a consequence, SAGE tags specific for the oocyst (sporozoite) stage were also expressed in the Day-4 population (Fig. 4A). For example, SAGE tags corresponding to the mRNA encoding the *Toxoplasma *homolog to the *Cryptosporidium *oocyst wall protein(s) [52] were only found in the oocyst and Day-4 libraries, as were SAGE tags for a recently described sporozoite surface antigen [53] and the sporozoite-specific superoxide dismutase [54]. Thus, parasites emerging after five divisions in the sporozoite-infected cell retain more of the sporozoite mRNA pool than was earlier anticipated [13], suggesting that the genetic program established during sporulation of the oocyst stage in the environment may have a significant influence on the initial phase of a primary *Toxoplasma *infection.

### Bradyzoite development begins with the post-growth shift at Day-7 following sporozoite inoculation and is accompanied by progressive changes in gene expression

We have previously reported that a slowing of the VEG strain tachyzoite replication rate roughly 20 division cycles after sporozoite-infection of HFF cells precedes the immediate onset of bradyzoite development [13]. We predicted that mRNA pools at Day-7 in the developmental series would reflect the transition of these parasites between rapidly-growing parasites at Day-6 and the full emergence of bradyzoites by Day-15, and in the pH-shifted populations. For this reason, Day-7 post-sporozoite populations were critical to our analyses of the developmental transcriptome because they represent the earliest observation of the growth-shifted population that ultimately commits the parasite to bradyzoite differentiation. In characterizing the differences between Day-7 and Day-6 mRNA pools, it was evident that nearly 1,181 SAGE tags in the Day-7 population were altered from the Day-6 population. Approximately 750 and 420 of all Day-7-tags were up-regulated or down-regulated (± 2.5-fold), respectively, when compared to the frequency of SAGE tags in the Day-6 population. From the up-regulated tags in the Day-7 library, 490 tags were completely absent from the Day-6 library, and conversely, 251 tags that were down-regulated in the Day-7 population were also absent in Day-6. Figure 4C displays all SAGE tags that were observed to be Day-7-specific in comparison to the other five developmental libraries without regard to the three laboratory strains.

Congruent with the observed induction of the slow growth that leads to initiation of the bradyzoite development pathway, levels of some mRNAs encoding proteins necessary for nuclear replication and cell division were reduced in the Day-7 population. For example, the mRNA encoding PCNA1 (down 3-fold) [55] and the mRNAs for DHFR-TS and the adenosine transporter – each providing dTTP and dATP, ultimately to be used in DNA synthesis [56] – were down-regulated in the Day-7 population. Consistent with the reduced replication rate at Day-7, both Rab5 and Rab11, which were up-regulated in the Day-6 population, were down-regulated 4- and 3-fold, respectively, suggesting a reduced need for endocytosis and cytokinesis at this stage of development [57,58]. We also observed reduced levels of mRNAs encoding many of the energy-related proteins up-regulated in the rapidly growing Day-6 population. For example, we observed a distinct reduction in the level of mRNAs encoding fructose-1,6-bisphoshatase [59], GAPDH and 3-phosphoglycerate kinase. We also observe the down-regulation of mRNAs encoding several molecules in mitochondrial electron transport including cytochrome b (down 8-fold) and cytochrome c oxidase (down 6-fold).

The presence together of tachyzoite and some bradyzoite specific markers in the Day-7 population is likely reflective of their position between two distinctly different stages of development, the rapidly replicating tachyzoite and the slowly growing or growth arrested bradyzoite. However, there is little expression of the most well studied bradyzoite genes (Day-7 vs. pH-shift libraries r = 0.0888, Table 2) and this is consistent with earlier measurements of BAG1 protein in these populations (2% BAG1;[13]). Marker co-expression extends to the tachyzoite-specific surface antigen SAG1 (up 20-fold) [60,61] and the mRNA encoding MIC10 (up 8-fold), while the increased level of the mRNA encoding the ROP4 protein (up 4-fold) is consistent with reports of higher levels of this transcript in the bradyzoite stage [22]. The appearance of the mRNA encoding hsp90 in the Day-7 population, which has previously been demonstrated to be up-regulated in the slow or non-replicating bradyzoite form [62], suggests that this mRNA may be an early bradyzoite marker. It is intriguing that drugs that target hsp90 prevent bradyzoite differentiation in laboratory strains [62], indicating an important role for this factor in initiating bradyzoite development.

Based on SAGE data, parasites from Day-15 post-sporozoite infections were a mixture of tachyzoite and bradyzoite forms with reduced growth rates [13] that express additional bradyzoite markers. Thus, for the first time in this population, we observed SAGE tags corresponding to bradyzoite markers SAG4.2 [34] and ENO1 [19], and although tachyzoite genes such as SAG1 and SAG2 [16] remain present, they show a decreased expression of these mRNAs compared to Day-7 parasites, indicating that the switch to the bradyzoite stage is continuing to progress. Figure 4D shows bradyzoite-specific genes that are shared between Day-15 and pH-shifted populations. The pH-shifted parasite populations represent the best-defined bradyzoite phenotype we can achieve in tissue culture host cells. These mRNA pools were representative of a definitive phenotypic change in the growth and development of VEG strain parasites along the life-cycle continuum in the intermediate host [13]. Similar to the significant changes observed in the Day-7 to Day-15 transition (15%), we observed that 1,441 of the SAGE tags analyzed were altered by more than 2.5-fold from the Day-15 population to the mature bradyzoite induced by alkaline stress (Fig. 4D). In this comparison, 695 tags analyzed were up-regulated in the pH-shifted library, while 746 were down-regulated. As expected, genes associated with cellular growth continue to be down-regulated in the pH-shifted library, consistent with the arrest of growth in these populations and the G1/G0 state of parasites from in vivo cysts [14,26]. We observed a dramatic disappearance of the mRNA encoding the inner membrane complex protein IMC-1 [63-65], which, notably, was also absent from the growth arrested sporozoite stage. Levels of mRNAs encoding other microtubule and/or cytoskeletal proteins including β tubulin [66,67] and various myosin precursor(s) were also down from the levels observed in the Day-7 and Day-15 parasite populations. Genes encoding DHFR-TS and PCNA1 and 2, which are up-regulated in the Day-4 and -6 populations, were also expressed at lower levels in the pH-shifted population as they were in the Day-7 population.

The unique list of up-regulated genes in the Day-15/pH-shifted libraries (graphed in Fig. 4D) included most of the well studied markers of bradyzoite differentiation. The *Toxoplasma *mRNA encoding lactate dehydrogenase 2 (bradyzoite-specific) [68], was present (while lactate dehydrogenase 1 was reduced 8-fold), as was mRNAs encoding Hsp30/BAG1 (7-fold up), p18 (SAG4.2) bradyzoite surface antigen, and *Toxoplasma *enolase 1, with all of these mRNAs substantially higher in the pH-shifted population when compared to Day-15. NTPase 1 and 3 mRNAs were down-regulated while a novel mRNA encoding a bradyzoite-specific NTPase (brady-NTPase) was observed to be dramatically up-regulated. The gene encoding Brady-NTPase (chr. X, TGG_994683) contains a single exon of 645 amino acids (~70 kDa) and is related (41% identity) to other well studied NTPases [69,70]. SAGE tag frequencies for Brady-NTPase indicates that it is an abundant mRNA in the pH-shift library, and we have confirmed mRNA expression by RT-PCR and the presence of bradyzoite-specific cis-elements in the 5'-intergenic region flanking this novel NTPase (Radke and White, unpublished). The NTP3 promoter contains tachyzoite-specific cis-elements [71], and thus Brady-NTPase may represent a bradyzoite-specific isoform of this enzyme.

### Laboratory adapted parasite strains possess gene expression that is characteristic of specific points in the parasite developmental pathway

Comparisons of SAGE datasets from the Type I, II and III laboratory strains with the VEG sporozoite-developmental series described above demonstrate correlations that may be associated with the capacity of each strain to differentiate. As expected, few of the genes specifically regulated in sporozoite/Day-4 populations were shared with the three tissue culture-adapted strains (Fig 4A), especially Type I and II strains. By contrast, a significant number of genes up-regulated in the Day-7 post-sporozoite populations were also found to be expressed at higher levels in all three lab strains (Fig 4B). Comparative similarity in mRNA pools was observed to rapidly diverge when the laboratory strains were compared to populations prior to and following the initiation of bradyzoite differentiation (Day-15 and pH-shifted populations). There is a striking correlation in the specificity and expression levels of a set of up-regulated SAGE tags (120 tags, Fig. 4B) specific for the Day-6 post-sporozoite populations that are also expressed in the RH laboratory strain (0.785 correlation), but largely not observed in the other developmental populations or the other laboratory strains. This unique relationship in gene expression may reflect a shared biology; Day-6-VEG populations, like RH parasites, lack any evidence of sporozoite or bradyzoite mRNA expression and grow with a similarly fast doubling time [13,26]. In contrast, SAGE libraries constructed from Type II-Me49B7 and Type III-VEGmsj parasites do not have elevated Day-6 SAGE tags (0.343 and 0.049 correlations, respectively, Table 2), but unlike the RH/Day-6 datasets, SAGE tags corresponding to bradyzoite genes are found in these libraries (e.g. basal levels of SAG4.2, Brady-NTPase and ENO1 are detected; see Fig. 4D). Baseline expression of bradyzoite genes might be the result of a minor population that had differentiated, although we have not observed this population in Type III-VEGmsj using known bradyzoite markers (not shown). Interestingly, elevated basal expression of bradyzoite genes has also been recently detected in the microarray analysis of avirulent laboratory strains that are developmentally competent, but presumed to be growing as tachyzoites (Boyle and Boothroyd, unpublished). The basal expression of bradyzoite genes that we and others have observed is likely to be related to the greater capacity of VEGmsj and Me49B7 parasites and other competent strains to enter the bradyzoite developmental pathway in vitro (and in animals), and thus the mRNA patterns in laboratory-adapted strains appear to mirror characteristics from the natural development pathway with their comparative position with respect to Day-7 post-sporozoite populations. Strains of which the mRNA profile is more consistent with development patterns that are earlier than Day-7 parasites, such as RH, may be more removed from bradyzoite differentiation, while gene expression profiles consistent with populations that have progressed beyond Day-7 (i.e. evidence of basal bradyzoite gene expression) may indicate that the parasites are primed to enter the bradyzoite developmental pathway. The level of annotation in the RH/Day-6 mRNA cluster is low (~80% are unique), providing relatively few clues to the biochemical pathways that are transiently expressed during VEG development but in RH are permanently activated. It is notable, however, that SAGE tags in this cluster correspond to ROP1 transcripts given that a minor QTL associated with RH acute virulence has been closely mapped to this locus in the *T. gondii *genome [72]. In addition, DHFR-TS is also found in this pool, which may be consistent with the well known relationship between DHFR and eukaryotic replication. It is likely not a coincidence that a single QTL associated with parasites that display the characteristic rapid cell cycle rates of Type I strains has been preliminarily mapped to the DHFR-TS locus on chromosome XII (Jerome, Behnke and White, unpublished results). Thus, the 120 Day-6/RH-specific SAGE tags may represent an important mRNA group that is associated with the virulent and rapidly growing RH phenotype, and the down-regulation of these mRNAs during development adds support to the concept that developmental gene expression in *T. gondii *determines the observed stage-specific phenotypes that change in a predetermined hierarchical order [23].

## Conclusion

**Figure 5:**
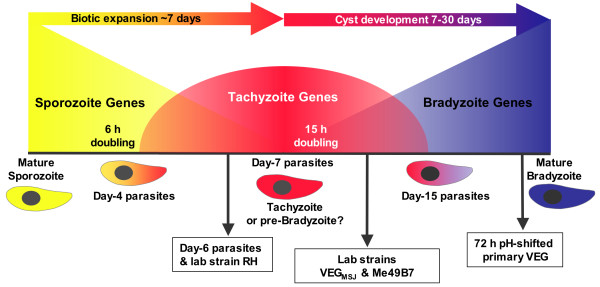
**Summary of the gene expression patterns associated with sporozoite-initiated development**. The relative timeline of sporozoite to bradyzoite development is indicated at the top of the diagram. Sporozoite- and bradyzoite-specific gene expression clearly demarcates these stages, while tachyzoite-specific patterns are more fluid with respect to developmental phenotypes. Gene expression patterns in Day-6 and RH parasites are highly correlated; however, the maximum tachyzoite antigen expression based on overall SAGE tag frequencies was observed in the Day-7 population. All laboratory strains are placed in the timeline on the basis of a best-fit criterion. Alkaline-shifted parasites (pH-shifted) express all the known bradyzoite genes, and are therefore considered to be nearly identical to mature bradyzoites in this diagram.

This report describes the first large-scale investigation of the *Toxoplasma *transcriptome during development in the intermediate life cycle. This comprehensive SAGE database offers a broad view of gene expression in primary as well as laboratory adapted parasite populations and defines fundamental changes in mRNA pools that will serve as a comparative base for future functional genomic studies. The emerging view from this study of gene organization and global mRNA expression demonstrates that mRNA classes based on abundance, function or co-regulation are dispersed throughout the chromosomes, which is consistent with a model where gene-specific mechanisms involving trans-acting factors are responsible for regulating mRNA levels in this protozoon. Experimental evidence shows that Apicomplexan protozoa promoters have a bipartite organization of basal and cis-elements [73] suggesting that transcriptional initiation in these parasites follows principles similar to other eukaryotes. It is noteworthy that ~60% of the factors necessary for general POL II transcriptional control in other crown group eukaryotes have now been found in Apicomplexa genomes [45]. Unfortunately, only a handful of *Toxoplasma *(or Apicomplexan) promoters have been investigated to identify intergenic regions that contain cis-element sequences [51,71,74-79] and only one gene-specific transcription factor in any Apicomplexan protozoan has been validated [80]. There is reason to believe that transcription in these parasites will have unique features. Unlike animal cells, where constitutive mRNA expression is a dominant feature, our studies show that transcription in *Toxoplasma *is considerably more dynamic with large numbers of mRNAs exclusively expressed in a single developmental stage. This phenomenon is consistent with the "just-in-time" concept put forth from studies of *Plasmodium *[47], where more than 80% of the transcripts monitored in microarray experiments were regulated, with most having a peak expression within a single timeframe in parasite development. It is plausible that this strategy for regulating gene expression in the Apicomplexa results in the unusual composition and changing complexity of *Toxoplasma *mRNA pools. Our results also demonstrate that gene expression leading to abundant mRNA levels in these parasites is focused on a select group of genes that have evolved with the adaptation of these protozoa to a parasitic life style and within the context of unique host-parasite relationships. Thus, it seems likely that gene-specific transcriptional mechanisms will be divergent in these parasites when compared to other well studied eukaryotic models and this view is supported by recent searches for general transcription factors in these parasites [46]. Gene expression associated with the major developmental stages studied here indicates that critical phenotypic transitions (i.e. changes in growth and developmental stage) are strongly influenced by changes in mRNA levels, and in those cases where data were available on protein changes, the mRNA levels were observed to move in similar directions. This too is consistent with the high degree to which protein and mRNAs levels correlate in the related parasite, *P. falciparum *[81]. Altogether these data do not rule out a role for post-transcriptional mechanisms in this parasite, but rather demonstrate that changes in mRNA levels play a significant role in regulating critical developmental transitions in this parasite. A summary of the overall gene expression patterns in sporozoite-initiated development is illustrated in Figure 5. It is significant that sporozoite gene expression influences early stages of the intermediate life cycle, given the similarities in gene expression between sporozoites and Day-4 emergent parasites. It is also noteworthy that tachyzoite gene expression, which is first detected in emergent parasites, remains strongly expressed in the slowly growing Day-7 parasites and is present in early bradyzoite, mixed populations (*i.e*. Day-15 parasites). Thus, tachyzoite gene expression was not confined to a specific developmental phenotype, and by itself was a poor gauge of a parasite's position within the intermediate life cycle. This may explain why laboratory strains considered to be tachyzoites based on antigen expression differ greatly in their replication rate and capacity to form bradyzoites. In fact, baseline expression of bradyzoite genes in VEGmsj and Me49B7 appears to be a more reliable predictor of the capacity of these strains to form bradyzoites, while the absence of this pattern in the RH strain is well correlated with developmental incompetence. Based on this principle, it is intriguing to speculate that expression in the highly virulent RH parasites of mRNAs also uniquely found in rapidly growing Day-6 parasites influences the characteristic resistance of RH and other Type I strains to form tissue cysts [72,82]. The Type I parasites may harbor a mutation that alters a critical regulatory mechanism controlling the capacity of these parasites to progress into the bradyzoite developmental pathway and this is reflected in the sustained (rather than transient) expression of a cluster of Day-6 genes in these parasites.

## Methods

### Parasite culture and isolation of total RNA

RH strain tachyzoites were maintained by serial passage in human foreskin fibroblasts (HFF) and these cells were cultured in Dulbecco's Modified Eagle Medium (DMEM, Gibco BRL, Grand Island NY) supplemented with 1% (v/v) newborn calf serum (Hyclone Laboratories Inc., Logan UT) as previously described [13]. To harvest total RNA for SAGE library construction, parasites were scraped from cultured cells, needle-passed and filter-purified from host cell debris using a 3 μM nucleopore membrane. Parasites were pelleted and total RNA was extracted from the pellet twice using 10 and then 5 ml of TRIzol according to the manufacturer's protocol (Gibco BRL, Rockville MD). Total RNA was also isolated from VEG strain oocysts that were obtained by sucrose flotation from cat feces as previously described [53,83].

### SAGE library construction

SAGE libraries were constructed from cDNA that was synthesized from 1 μg of total RNA using the Smart™ cDNA synthesis reagents (Clontech, Palo Alto, CA). Briefly, a biotinylated oligo-dT primer (5'-AAGCAGTGGTAACAACGCAGAGTAC(T)_30_VN-3' where V = A, C or G, and N = T, C, G or A) and Superscipt II reverse transcriptase (Life Technologies, Framingham MA) were used to make 1^st ^strand cDNA. Second-strand synthesis and cDNA amplification was completed by PCR using the Advantage-2 Polymerase Mix (ClonTech, Palo Alto CA), and a switching-primer (5'-AAGCAGTGGTAACAACGCAGAGTACGCGGG-3') in combination with the original biotinylated oligo-dT. Approximately 22 amplification cycles provided 10 μg of double-stranded cDNA used to construct(s) the library of SAGE tags according to standard protocols [27,29,84]. To improve the cloning efficiency in the final stage of construction, we separated SphI-digested DNA by cDNA size-exclusion chromatography (manual #235612, Stratagene, La Jolla, CA) using the Sepharose CL-2B matrix (450 bp minimum cutoff, Sigma-Aldrich, St Louis, MO). We have determined that this method will cleanly separate linear cDNA fragments from circular DNA that forms during concatemerization and are not linearized in the SphI digestion (and are therefore unclonable; Radke and White, unpublished). Concatemer fragments eluted from the column were collected in twelve 100 μl fractions and the 1 to 2 kbp fragments were isolated from fractions 4, 5 and 6 by overnight precipitation in ethanol at -20°C. Purified fragments were cloned into pZero plasmid vector (Invitrogen, Carlsbad CA) that had been linearized with SphI. Following electroporation of DH10B cells (Invitrogen, Carlsbad CA), transformed colonies were plated on low LB-agarose containing zeocin (50 μg/ml) and picked for sequence analysis by standard methods.

### SAGE tag extraction and construction of SAGE datasets

Delineation of sequence, extraction of SAGE tag information, frequency analyses and tag sequence annotation were all completed using Perl 5.6.1 [85] running in a UNIX RedHat 7.2 environment [86] with Perl scripts written and developed in this laboratory. Each cloned concatemer contains nucleotide sequence of repeating units of SAGE ditags, separated by a single NlaIII restriction endonuclease consensus sequence (CATG). We extracted SAGE tag sequences using CATG landmarks and the regular alternating 28 to 33 base nucleotide sequence defining each ditag. Ditag sequence was processed using software previously developed to extract individual SAGE tag information, record tag frequency and correct sequence error in the raw dataset by nearest neighbor analysis [87]. Tag frequencies for each library were normalized by multiplying the tag count by the ratio of adjusted library size, divided by the actual size – where the adjusted size was equal to 50,000 tags.

The resulting dataset was stored and organized using MySQL [88] with web-based access via the Apache web server [89]. Queries of raw SAGE tags or those corrected for sequencing error, normalized or annotated tags (as defined by BLAST score), may be performed at TgSAGEDB [31]. Queries to TgSAGEDB datasets result in an ordered list of SAGE tags ranked by frequency and library choice with individual frequencies displayed across columns for each of the nine SAGE libraries. Dynamic links within the page connect the individual to the position of each tag within the *Toxoplasma *chromosome maps [90] or within the assembled genomic contigs and linkage to ApiDots [36] via tag sequence cross-connects SAGE tags to the *Toxoplasma *EST collection. Each tag in the results page is linked to other possible positions in the *Toxoplasma *genome (the initial 2 × 2 choice limits this to ≤ 2 hits), or to nearest neighbor tags (which identifies putative SNPs) that differ by a single nucleotide. Tag clusters are displayed in the *Toxoplasma *genomic contigs via a defined bracket set by the user (2 kbp, 5 kbp or 10 kbp on either side of the specific tag chosen). A link to the 2 kbp genomic sequence immediately adjacent to a tag and in the same strand orientation is provided along with information on tBLASTx annotations (if any). A final link takes the individual to a gene ontology site where BLAST results may be reviewed with respect to GO assignments. From the *Toxoplasma *GO database the individual can link back to the SAGE results via related gene products in order to assess co-regulation of specific pathways.

### SAGE analysis

SAGE tags and their normalized frequencies were imported into GeneSpring 7.2 (Agilent Technologies Inc., Palo Alto, CA) used for additional analyses including the generation of standard correlations between SAGE library datasets. GeneSpring export file(s) can be downloaded from the TgSAGEDB website [31]. Gene expression comparisons across developmental and strain libraries were performed in GeneSpring 7.2 by filtering gene lists by expression level in the 2 × 2 dataset normalized with ratio mode (signal/control). These gene lists were compared by clustering analysis using Pearson correlation as a similarity measurement. Standard k-means clustering using 100 iterations and k = 7 (k>8 resulted in progressive separation of genes into redundant clusters) generated gene lists that were highly similar to those generated by the change in fold expression (results based on fold change are shown in Fig. 4).

In order to annotate SAGE tag sequence(s), and assign gene function where available, tag sequences were compared to the 10 × *Toxoplasma gondii *genome obtained from the Me49B7 strain. For exact sequence matches in either strand (+ or -), the matching genomic contig number, sequence position in the contig and + or - strand orientation were recorded. Since the tag sequences have a greater bias toward the 3' end of the mRNA, we extracted 2,000 nucleotides directly 5' of each SAGE tag in the contig in order to associate a larger portion of the potential coding sequence with each tag. This dataset was blasted locally against the non-redundant database of protein sequences (nr, NCBI) using the BLASTall/BLASTx program. Each sequence was annotated using the BLAST alignment with the lowest expected value (≤ 1 × 10^-6^) in those alignments where the reading frame was in the positive orientation. Additionally, the ten best alignments that met these criteria were also associated with the sequence in order to provide expanded annotation information. To estimate the number of unique transcripts in the SAGE dataset (accounting for multiple polyA+-additions), we used cap3 [91] to assemble the 2,000 nucleotide sequences 5' to each tag. Total assembled contigs and singletons (individual sequences that did assemble into any one contig) were used to predict the number of unique transcripts in the SAGE dataset.

To determine the presence and level of potential antisense transcription, SAGE tags that matched the genome once and had a sum frequency of = 2 across all libraries were matched to predicted gene annotation. Four distinct gene prediction datasets (GeneFinder, TwinScan, TigrScan and Glimmer) were available for comparison from ToxoDB [25,92]. In these comparisons, for each predicted gene, tags matching the (+) strand were defined as 'sense' and those matching on the (-) strand 'antisense'; and frequencies were recorded separately.

### SNP and polyA+ analysis

SNP analyses were completed using the EMBOSS program 'fuzznuc' [93,94] to identify individual SAGE tag sequences with a 1 nucleotide mismatch where present in the dataset. A single base mismatch across tag sequence(s) from one or more strain types was defined as a potential SNP. To test whether these mismatched bases would accurately identify the presence of a real SNP, predicted tags where the single-base mismatch occurred in the consensus sequence for a unique restriction endonuclease were identified and flanking primers used to amplify these regions from genomic DNA by polymerase chain reaction (PCR). Two such Type II strain tag sequences were selected for this analysis: (1) the SUL1 (MnI) SAGE tag, CATGACATCGAGGA (present at nucleotide position 332,392 in genomic contig TGG_994387), and (2) the AcI I-SNP tag CATGCTCCGCTACA (nucleotide position 39,009, contig TGG_994300). These were amplified separately from Type I (RH), II (Me49B7) and III (VEG) strain genomic DNAs (MnI; F-AAGAGTGTGCTTTGGTGCCTTATTG and R-ACACTCCGCGATTGCACACAC, and AcI; F-GCCTCGCACAATCACTGGTTTGAC and R-CGGAAGAGGAAGAAGTTGCCG). Amplified products were purified by spin column according to standard protocols (Stratagene, La Jolla, CA) and digested with the appropriate restriction enzyme (AcI or MnI), and fragments were resolved by 6% polyacrylamide gel electrophoresis.

To assess differential polyA addition to mRNAs encoding GRA7, total RNA was isolated by spin column (QIAGEN, Alameda, CA) from each of the three strain types (as described above) and subjected to 3'-RACE [95] using a modified oligo-d(T) primer, containing attB2 recombinational cloning sequence, and a GRA7-specific primer containing the attB1 sequence [96] 5'-GGGGACCACTTTGTACAAGAAAGCTGGGTC(T)_30_VN-3' and GRA7 5'-GGGGAGAAGTTTGTACAAAAAAGCAGGCTTCCGAGCAAGAGGTGCCTGAATCAGG-3'). Amplified products from each PCR reaction were resolved by 6% polyacrylamide gel electrophoresis. RACE products from three distinct cycle profiles (15, 20, 25 cycles) were compared to verify that the relative abundance of the amplified products was not influenced by cycle number (not shown). Additionally, amplified products were cloned by recombination into p221DONOR vector (Gateway™ Invitrogen Co., Carlsbad, CA) and sequence analyses used to verify the GRA7 product. To demonstrate the distance from the NlaIII restriction endonuclease consensus sequence (in the nearest upstream SAGE tag) to the polyA^+^-addition site(s), 3'-RACE products were compared to that predicted using the distance between this NlaIII site and the GRA7-specific primer. All predicted fragment lengths were compensated to include the 90 additional nucleotides contributed by the attB1 and attB2-oligo-d(T)_30 _primer sequences incorporated into the RACE fragments.

## Authors' contributions

This study was conceived and designed and the manuscript draft written by MWW, JRR and MB. JR constructed most of the SAGE libraries and analyzed differential polyA-addition. AJM, MB, JRR, DSR and MWW designed the SAGE database and web site and analyzed the SAGE database with respect to normal distribution, annotation, frequency and developmental specificity.

## Figures and Tables

**Table 1:** Global statistics

**SAGE Tag Datasets**	**Total**	**10 × Gen. Match**		**No Gen. Match**		**Annotated Seq.**		**EST Match**	
SAGE tag counts: ALL	290,062	262,029	[90.3%]	28,033	[9.7%]	128,519	[44.3%]	207,625	[71.6%]
Unique SAGE tag counts: ALL	38,263	24,932	[65.2%]	13,331	[34.8%]	5,910	[15.4%]	8,709	[22.8%]
AVG FREQ SAGE tag counts: ALL	7.6	10.5		2.1		21.7		23.8	
									
SAGE tag counts: 2 × 2	202,472					102,619	[50.7%]	163,283	[80.6%]
Unique SAGE tag counts: 2 × 2	8,188					2,038	[24.9%]	3,560	[43.5%]
AVG FREQ SAGE tag counts: 2 × 2	24.7					50.4		45.8	

10 × Gen. = 10 × coverage of the genome sequence for reference strain Me49B7

**Table 2:** Pairwise comparisons of normalized tag frequencies for all nine SAGE libraries.

**Library**	**SP**	**D4**	**D6**	**D7**	**D15**	**pH**	**MSJ**	**RH**	**ME49**
**SP**	1	**0.728**	0.0229	0.121	0.112	0.0496	0.0557	0.022	0.0205
**D4**	**0.728**	1	0.0625	0.275	0.271	0.109	0.137	0.0554	0.0476
**D6**	0.0229	0.0625	1	0.0604	0.0781	0.0358	0.0486	**0.785**	0.343
**D7**	0.121	0.275	0.0604	1	0.32	0.0888	0.151	0.066	0.0809
**D15**	0.112	0.271	0.0781	0.32	1	0.239	0.181	0.0536	0.0623
**PH**	0.0496	0.109	0.0358	0.0888	0.239	1	0.119	0.0281	0.0273
**MSJ**	0.0557	0.137	0.0486	0.151	0.181	0.119	1	0.0348	0.0444
**RH**	0.022	0.0554	**0.785**	0.066	0.0536	0.0281	0.0348	1	0.276
**ME49**	0.0205	0.0476	0.343	0.0809	0.0623	0.0273	0.0444	0.276	1

Sp = sporozoite/oocyst, D4, -6, -7, -15 = days post-sporozoite infection, pH = 72 h culture of VEG strain in 8.2 pH-medium, and laboratory strains: MSJ = Type III VEG, RH = Type I, ME49 = Type II
